# Impact of beam configuration on VMAT plan quality for Pinnacle^3^Auto-Planning for head and neck cases

**DOI:** 10.1186/s13014-019-1211-6

**Published:** 2019-01-18

**Authors:** Anne Richter, Florian Exner, Klaus Bratengeier, Bülent Polat, Michael Flentje, Stefan Weick

**Affiliations:** Department of Radiation Oncology, University of Würzburg, Josef-Schneider-Str. 11, 97080 Würzburg, Germany

**Keywords:** Auto-planning, VMAT, Single arc, Double arc, Full arc, Partial arc, Plan comparison

## Abstract

**Background:**

The purpose of this study was to compare automatically generated VMAT plans to find the superior beam configurations for Pinnacle^3^ Auto-Planning and share “best practices”.

**Methods:**

VMAT plans for 20 patients with head and neck cancer were generated using Pinnacle^3^ Auto-Planning Module (Pinnacle^3^ Version 9.10) with different beam setup parameters. VMAT plans for single (V1) or double arc (V2) and partial or full gantry rotation were optimized. Beam configurations with different collimator positions were defined. Target coverage and sparing of organs at risk were evaluated based on scoring of an evaluation parameter set. Furthermore, dosimetric evaluation was performed based on the composite objective value (COV) and a new cross comparison method was applied using the COVs.

**Results:**

The evaluation showed a superior plan quality for double arcs compared to one single arc or two single arcs for all cases. Plan quality was superior if a full gantry rotation was allowed during optimization for unilateral target volumes. A double arc technique with collimator setting of 15° was superior to a double arc with collimator 60° and a two single arcs with collimator setting of 15° and 345°.

**Conclusion:**

The evaluation showed that double and full arcs are superior to single and partial arcs in terms of organs at risk sparing even for unilateral target volumes. The collimator position was found as an additional setup parameter, which can further improve the target coverage and sparing of organs at risk.

## Background

Today intensity-modulated radiation therapy is a widely used clinical treatment modality in many countries utilized to achieve improved target dose conformity and better sparing of critical structures [1]. During an iterative process, the objective parameters are adjusted to obtain a clinically acceptable dose distribution. The resulting plan quality is user dependent due to individual experience and optimization strategies. Several approaches for automated treatment planning were developed to overcome this limitation like multi-criteria optimization [2, 3], knowledge-based treatment planning [4], or automated treatment planning in Pinnacle^3^ (Philips Radiation Oncology Systems, Milpitas, CA, USA) [5, 6]. Several studies have compared automatically with manually generated plans for different entities using intensity or volumetric modulated arc therapy (VMAT). Most of them concluded that Pinnacle^3^ Auto-Planning offers similar target coverage and improved sparing of organs at risk (OARs) [5, 7–13].

The purpose of this study was to compare automatically generated VMAT plans to determine a superior beam arrangement as preset for Pinnacle^3^ Auto-Planning. Two evaluation methods were used, one of them was newly developed for this study.

## Methods

For this retrospective planning study, the influence of VMAT beam configuration on plan quality was investigated for 20 patients treated for head and neck cancer. Table 1 gives an overview of patient and treatment characteristics. Bilateral and unilateral planning target volumes (PTV) were chosen to consider different shapes of target volumes.

**Table 1:** Overview of patient population

Case	Disease type	Treatment concept	Fractionation Scheme Dose D_95_ in Gy	Fractions	Side location
1	SCC, Hypopharynx	Primary RCT	59,4/66/69,3	33	bilateral
2	SCC, Oral cavity	Adjuvant RT	54/66	30	bilateral
3	SCC, Oropharynx	Adjuvant RT	54/66	30	bilateral
4	SCC, Oral cavity	Adjuvant RCT	54/66	30	bilateral
5	SCC, Oropharynx	Adjuvant RCT	60/66	30	bilateral
6	SCC, Oral cavity	Adjuvant RCT	54/66	30	bilateral
7	SCC, Oropharynx	Adjuvant RCT	54/66	30	bilateral
8	SCC, Oral cavity	Adjuvant RCT	54/66	30	bilateral
9	CUP, Oropharynx	Adjuvant RT	52,7/65,1	31	bilateral
10	SCC, Oral cavity	Adjuvant RCT	54/66	30	bilateral
11	SCC, Oropharynx	Adjuvant RT	54/66	30	unilateral
12	SCC, Oropharynx	Adjuvant RCT	54/66	30	unilateral
13	SCC, Oropharynx	Adjuvant RCT	60/66	30	unilateral
14	Undifferentiated orbital cancer	Adjuvant RT	60/66	30	unilateral
15	SCC, external auditory canal	Adjuvant RT	54/66	30	unilateral
16	SCC, occipital skin	Adjuvant RT	54/66	30	unilateral
17	SCC, Oropharynx	Adjuvant RCT	54/66	30	unilateral
18	SCC, Nasal cavity	Adjuvant RT	54/66	30	unilateral
19	SCC, Oral cavity	Adjuvant RCT	60/66	30	unilateral
20	NHL of the parotid gland	Adjuvant RT	40	20	unilateral

All except one patient (Case #20) were treated with a simultaneously integrated boost technique with 2–3 dose levels

*CUP* Cancer of Unknown Primacy, *RCT* Radio-Chemo Therapy, *RT* Radiotherapy, *SCC* Squamous Cell Carcinoma

Disease type, treatment concept, fractionation scheme and tumor side location are described for all 20 patients

### Beam configuration

The Pinnacle^3^ Auto-Planning engine (Version 9.10) was used for optimization of VMAT plans. Treatment was planned for an Elekta Synergy Platform® equipped with Agility Head (Elekta Oncology Systems, Crawley, UK). The VMAT beam configuration was varied (overview and naming see Table 2). For each patient, the clinically accepted treatment plan was evaluated by a physician and served as reference (V2_C15_). Different arc types were compared: one single arc (V1_C15_), two single arcs (2V1_C15_) or one double arc (V2_C15_). Two single arcs can be directly created by the user before optimization (2V1_C15_) while both arcs are treated equally during optimization. In contrast, one double arc is generated by the system during optimization after only one arc was initially defined by the user (V2_C15_). The influence of arc length (full or partial arcs) was evaluated for 10 cases with unilateral located targets (see Table 1). Start and stop angles of partial arcs were set depending on the treated side: 300° to 179° for left sided targets and 181° to 60° for right sided targets.

**Table 2:** Overview of beam configurations and list of comparisons. Varying beam configurations are displayed on the left part: Single, double and two single arc types, full and partial arc length and different collimator positions were defined and used for comparison. The corresponding list of comparison between the different beam configurations is displayed on the right part

Name	Arc type	Arc length	Collimator position	List of comparisons
V1_C15_	Single arc	Full	15°	1. V2_C15_ vs. V1 _C15_
V2_C15_	Double arc	Full	15°	2. V2_C15_ vs. 2V1_C15_
V2_C15_Part_	Double arc	Partial	15°	3. V2_C15_ vs. V2_C15_Part_
V2_C15_	Double arc	Full	15°	4. V2_C15_ vs. V2_C40_
V2_C40_	Double arc	Full	40°	5. V2_C15_ vs. V2_C60_
V2_C60_	Double arc	Full	60°	6. V2_C15_ vs. 2V1_C15_60_
2V1_C15_	Two single arcs	Full	15°, 15°	7. V2_C15_ vs. 2V1_C15_345_
2V1_C15_60_	Two single arcs	Full	15°, 60°	8. 2V1_C15_60_ vs. 2V1_C15_345_
2V1_C15_345_	Two single arcs	Full	15°, 345°	

Note: Single arc with collimator 15° (V1_C15_), Full double arc with collimator 15° (V2_C15_), partial arcs (V2_C15_Part_), Two single arcs with different collimator positions (A and B) (2V1_CA_CB_), Partial arc with collimator 15° (V2_C15_Part_)

The impact of collimator setting was explored for two single arcs and double arcs separately. For double arc technique, the impact of different collimator positions was additionally investigated (V2_C15_, V2_C40_ and V2_C60_). The chosen collimator angles (15°, 40°, 60°) are restricted to the first quadrant. The second quadrant is covered by the opposed gantry position in a course of a full rotation. The third and fourth quadrant would be covered by switching X1 and X2.

For two single arcs, the combination of different collimator positions was examined. The combination of collimator positions 15° with 345° was compared to 15° with 60°.This resulted in the comparisons as listed in Table 2 (right column).

### VMAT optimization

The goals for PTVs and OARs were defined based on the evaluation parameter set (see Table 3). Planning structures were created based on desired target coverage and OAR sparing. For simultaneous integrated boost techniques, the difference of target volumes and planning organs at risk was calculated to avoid overlapping structures. The Auto-Planning goals of the clinically accepted treatment plan served as reference for the other techniques. For comparability, target and OAR goals, dose grid and structure definition remained the same for different beam configurations of one patient case. The dose distribution was calculated with collapsed cone algorithm, dose grid resolution was set to 2 mm and a control point spacing of 4° was used. In the Pinnacle^3^ Auto-Planning module advanced setting parameters were not changed (tuning balance between PTV and OARs 11%, dose fall-off margin 2,2 cm, hot-spot maximum goal 107%). Once the Auto-Planning run was completed, no further adjustment or re-optimization were performed.

**Table 3:** Example of evaluation parameter set for case #1

Structure	DVH Parameter		Dose Limit in relation to D_95_	Absolute Dose Limit in Gy
PTV1	D_95_		± 2%	69,30
	D_98_	>	0,95 D_95_	65,8
	D_min_	>	0,9 D_95_	62,4
	STD	<	3,3%	2,3
PTV2	D_95_		± 2%	66,00
	D_98_	>	0,95 D_95_	62,7
	D_min_	>	0,9 D_95_	59,4
	STD	<	3,3%	2,2
PTV3	D_95_	>	± 2%	59,40
	D_98_	>	0,95 D_95_	56,4
	D_min_	<	0,9 D_95_	53,5
	STD	<	3,3%	2,0
SpinalCanal	D _1cm_^3^	<		45
BrainStem	D _1cm_^3^	<		45
Left Parotid	D_66_	<		20
	D_mean_	<		26
Right Parotid	D_66_	<		20
	D_mean_	<		26
Larynx	D_05_	<		69
	D_05_	<		45
Mandible	D_05_	<		69
	D_10_	<		60
	D_50_	<		52
Neck	D_max_	<		40
Left Lens	D_05_	<		9
Right Lens	D_05_	<		9
Left OpticNerve	D_05_	<		50
Right OpticNerve	D_05_	<		50
Chiasm	D_05_	<		50
Pituitary	D_05_	<		50
Left InnerEar	D_05_	<		30
Right InnerEar	D_05_	<		30
Outline	D_Max_	<	1,15 D_95_	79,7

Definition of an evaluation parameter set for an integrated boost technique with three dose levels (PTV1, PTV2, PTV3). Requirements for target coverage and dose homogeneity are described by DVH parameters, relative and absolute dose limits scaled according to the prescription dose D_95_ of each PTV (top part). DVH parameters and absolute dose limits are listed for organs at risk (bottom part)

*D*_*Min*_ Minimum Dose, *D*_*Max*_ Maximum Dose, *D*_*Mean*_ Mean Dose, *STD* Standard deviation, *D*_*X*_ Volume X is covered by the dose value, *PTV* Planning target volume

Auto-Planning tries to mimic the decision-making process of an experienced operator. During Auto-Planning, individual optimization objectives, constraints and weights are automatically added and adjusted based on the user defined clinical goals. Structures of hot and cold spots are created to compensate for over and under dosage. The Auto-Planning module adjusts iteratively the objective set to best meet the planning goals. During optimization, a certain combination of cold and warm starts is performed. The resulting objective set represent in detail the desired shape of the dose volume histogram (DVH). Nevertheless, each optimization process for any patient-beam-configuration ends potentially in different sets of objectives, because Auto-Planning identifies weak points of the individual plans and tries to compensate them by appropriate additional objectives. For each plan, the composite objective value (COV) is calculated. The COV describes the weighted sum of quadratic deviations of the objective values and the related points in the dose volume histogram. In other words, the COV expresses how good the requirements were fulfilled (target coverage and OAR sparing). The optimization process minimizes COV.

### Dosimetric evaluation

Plan evaluation was based on two approaches: (i) scoring of evaluation parameters and (ii) indirect comparison of the COV using a new cross comparison method. The clinically accepted treatment plan served as reference (V2_C15_) for both evaluation methods. The comparison was done for a set of treatment plans for the same patient only.

For the scoring method, an evaluation parameter set was defined in consensus by the physicians of our institution and is considered as standard evaluation set (Table 3). For each patient, the dose limits were scaled according to the prescription dose (D_95_) in Table 1. Each plan was scored depending on how many requirements were met in the evaluation parameter set. All requirements were equally weighted. The sum of all scores was calculated for each plan. If a plan fulfilled the requirement, this plan achieved the score (see Table 4). If both plans met the requirement and the deviation was more than 1%, the better plan achieved the score. If the deviation was less than 1%, no trial achieved the score (tie). If both trials failed the requirement, the better plan achieved the score.

**Table 4:** Rules for the scoring method

Requirement met		Deviation (A vs. B)	Scoring
Plan A	Plan B		
true	true	≥ 1%	A or B (closer)
		< 1%	tie
false	false	≥ 1%	A or B (closer)
		< 1%	tie
true	false		A
false	true		B

If the deviation was less than 1%, no trial achieved the score (tie). If both trials failed the requirement, the better plan achieved the score. If a plan fulfilled the requirement (true), this plan achieved the score

If both plans met the requirement and the deviation was more than 1%, the better plan achieved the score

**Fig. 1:**
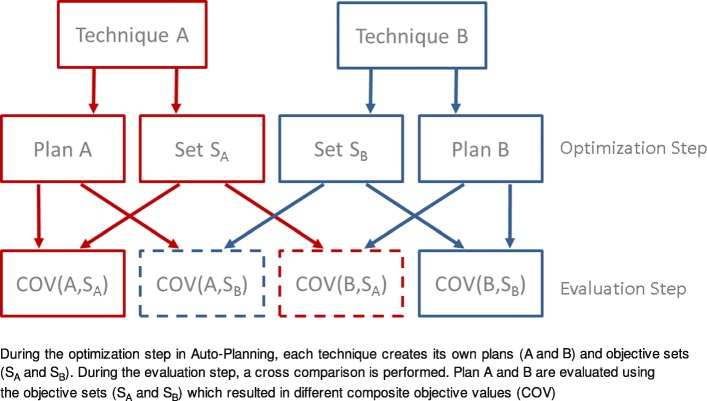
Flow chart of COV method

The second approach compared indirectly the COVs of two plans. The principle of cross comparison is illustrated in a flowchart in Fig. 1. During the optimization step, each technique A creates its own plan A and its corresponding set of objectives S_A_. For the evaluation step, the assessment results are expressed by COV(A, S_A_). As illustrated in Fig. 1, plan A (generated using technique A) is subjected to its own objective set S_A_, resulting in COV(A, S_A_). Similarly, plan B creates its own objective set S_B_, resulting in COV(B, S_B_). The values COV(A, S_A_) and COV(B, S_B_) cannot be compared directly because the COVs were calculated based on different objective sets (S_A_ and S_B_). Therefore we propose a cross comparison method; the measures S_A_ and S_B_ are mutually applied:

Technique A is allowed to set the conditions S_A_ for assessment of plan B leading to COV(B, S_A_). A symmetric situation is effected when also Technique B is allowed to set its valuation standard S_B_ resulting in COV(A, S_B_). Finally, each of both plans A and B is evaluated with two parameter sets (S_A_ and S_B_). As long as each plan wins only according to its *own* objective set, no decision can be made on which plan is superior (COV(A, S_A_) < COV(B, S_A_) and COV(B, S_B_) < COV(A, S_B_)). In contrast, if plan A wins according to *both* objective sets S_A_ and S_B_, it can clearly be regarded as the better technique: Plan A is better according to its own measure and it is better even if plan B sets the assessment scale. In other words, plan quality of plan A is superior to plan B, if both values of COV(A, S_A_) and COV(A,S_B_) are lower than COV(B,S_B_) and COV(B,S_A_). We denote “plan A is superior to plan B” as proposition “F_A > B_”; it is true, if

1 $$ \left[ COV\left(A,{S}_A\right)< COV\left(B,{S}_A\right)\cap \kern0.333em COV\left(A,{S}_B\right)< COV\left(B,{S}_B\right)\right] $$

Re-arrangement of Eq. 1 leads to:

2 $$ 1<\frac{COV\left(B,{S}_B\right)}{COV\left(A,{S}_B\right)}\cap \kern0.333em 1<\frac{COV\left(B,{S}_A\right)}{COV\left(A,{S}_A\right)} $$

An inversion of the left part of Eq. 2 results in

3 $$ \left[\frac{COV\left(A,{S}_B\right)\ }{COV\left(B,{S}_B\right)}<1<\frac{COV\left(B,{S}_A\right)}{COV\left(A,{S}_A\right)}\ \right] $$

which is a reformulation of F_A > B_.

The difference of scoring was tested using Wilcoxon matched-pair signed rank test. The level of significance was set at 0,05. Plan A is superior to plan B as soon as one of two evaluation methods shows a significant difference between the techniques.

Additional parameters were assessed to investigate how the beam arrangement affects the plan metrics: monitor units (MU), delivery time (T), Paddick conformity index (CI).

## Results

**Fig. 2:**
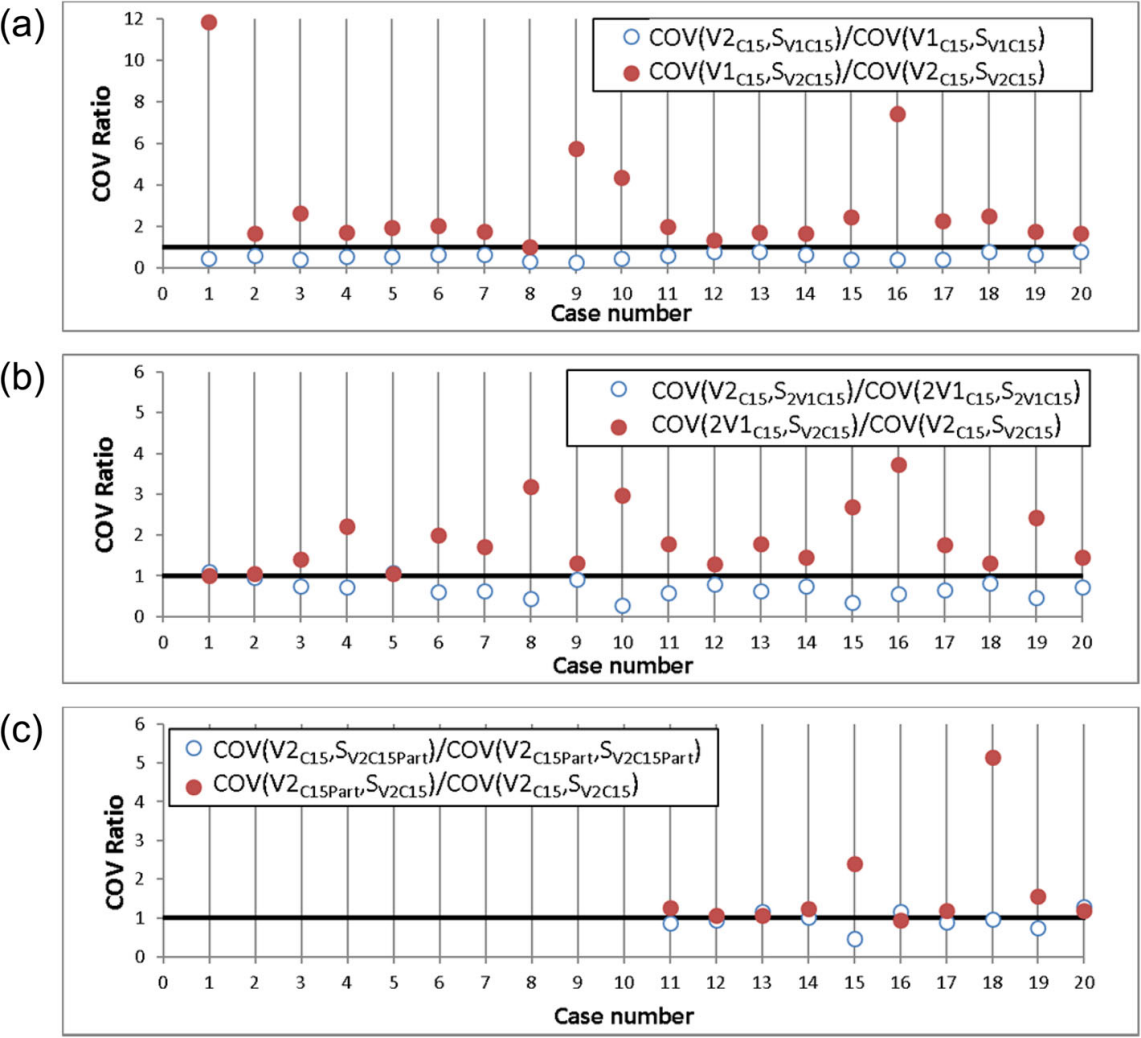
Comparison of plan quality with the COV method. Plan quality was evaluated and COV ratios for comparison #1–3 are shown. Plan quality of V2_C15_ is superior if the first COV ratio is below 1 (open circles) and the second COV ratio is greater than 1 (filled circles). **a** COV ratios for comparison #1: single arc (V1_C15_) and double arc (V2_C15_) for 20 patients. **b** COV ratios for two single arcs with collimator rotation (2V1_C15_) and double arc (V2_C15_) for 20 patients. **c** COV ratios for full (V2_C15_) and partial arcs (V2_C15_Part_) for 10 patients with unilateral target volumes

First, the plan quality was compared for single and double arc techniques (V1_C15_ vs. V2_C15_). The results of the Wilcoxon Rank test are listed in Table 5. Plan quality differed significantly between V1_C15_ and V2_C15_ with *p* < < 0,05 for both evaluation methods (scoring and COV based). Figure 2a shows the COV ratios, expressing that V2_C15_ is preferable to V1, if F_V2 > V1_ is true:

4 $$ {\mathrm{F}}_{V2>V1}=\left[\frac{COV\left(V2,{S}_{V1}\right)\ }{COV\left(V1,{S}_{V1}\right)}<1<\frac{COV\left(V1,{S}_{V2}\right)}{COV\left(V2,{S}_{V2}\right)}\ \right] $$

**Table 5:** Summary of plan quality

	Comparison of techniques	p-value		Superior technique
		COV based	Scoring based	
1	V2_C15_ vs. V1 _C15_	0,00009 *	0,00009 *	V2_C15_
2	V2_C15_ vs. 2V1_C15_	0,00012 *	0,00054 *	V2_C15_
3	V2_C15_ vs. V2_C15_Part_	0,0047 *	0,036 *	V2_C15_
4	V2_C15_ vs. V2_C40_	0,12	0,78	
5	V2_C15_ vs. V2_C60_	0,00078 *	0,0083 *	V2_C15_
6	V2_C15_ vs. 2V1_C15_60_	0,43	0,39	
7	V2_C15_ vs. 2V1_C15_345_	0,01 *	0,041 *	V2_C15_
8	2V1_C15_60_ vs. 2V1_C15_345_	0,0051 *	0,22	2V1_C15_60_

Techniques with different beam configurations were compared with regard to plan quality using COV and scoring method. The *p*-value of the Wilcoxon signed rank test shows if the techniques differed significantly and the superior technique is listed

*Significant differences are marked (*p* < 0,05)

The evaluation of COV showed a superior plan quality of V2_C15_ compared to V1_C15_ for all cases because the first term in eq. 4 is below 1 (open circles in Fig. 2a) and at the same time the second term greater than 1 (filled circles). Table 6 shows the change of plan metrics for V1_C15_ compared to V2_C15_. A decrease in delivery time (24% on average) and monitor units (− 9%) was observed. For V1_C15_ the conformity was reduced by 4% on average.

**Table 6:** Summary of plan metrics

	Comparison of techniques	ΔMU/Gy in %	ΔT in %	ΔCI in %
1	V2_C15_ vs. V1_C15_	−9,0 ± 2,3	−24,4 ± 12,4	−4,2 ± 3,9
2	V2_C15_ vs. 2V1_C15_	−4,9 ± 2,8	4,4 ± 13,0	−3,0 ± 2,7
3	V2_C15_ vs. V2_C15_Part_	−3,4 ± 2,3	−15,9 ± 16,2	−2,4 ± 3,7
4	V2_C15_ vs. V2_C40_	−1,8 ± 4,2	7,4 ± 23,8	−0,5 ± 1,3
5	V2_C15_ vs. V2_C60_	−0,3 ± 3,4	61,5 ± 63,8	−1,7 ± 2,1
6	V2_C15_ vs. 2V1_C15_60_	−1,5 ± 3,2	5,0 ± 35,5	−0,4 ± 2,1
7	V2_C15_ vs. 2V1_C15_345_	−1,5 ± 2,9	29,2 ± 12,0	−1,5 ± 2,5

Techniques with different beam configurations were compared with regard to plan metrics. The deviation of monitor units (MU), delivery time (T) and Paddick Conformity Index (CI) were calculated between each plan version and the reference plan (V2_C15_). For all patients, the mean and standard deviation are shown for comparisons #1–7

In the second comparison, it was tested whether better plan quality can be achieved by a double arc or two single arcs (V2_C15_ vs. 2V1_C15_). The *p*-values in Table 5 indicate that V2_C15_ and 2V1_C15_ plans are significantly different. The results of the COV method are shown in Fig. 2b. Improved plan quality was observed for the double arc technique V2_C15_ in 18 out of 20 (90%) cases. The difference of COV values is minimal for case numbers 1 and 5. Similar plan quality could be achieved for these cases. The monitor units decreased by 5% on average while the delivery time increased by 4% on average. For 2V1_C15_ the conformity was reduced by 3% on average.

In the third comparison, the influence of arc length on plan quality was investigated for full and partial arcs (V2_C15_ vs. V2_C15_Part_). A significant difference was found between V2_C15_ and V2_C15_Part_. The graphical illustration of COV ratios is shown in Fig. 2c. In 6 out of 10 cases the plan quality was better if a full gantry rotation was allowed during optimization. Similar plan quality could be achieved for cases numbers 13, 14 and 20 using full and partial arcs. For V2_C15_Part_, delivery times were shorter (16% on average) and conformity was reduced by 2,4% on average. The change in monitor units was minimal (− 3%).

The fourth and fifth comparison explored how collimator setting affects plan quality. Based on the COV evaluation, a double arc technique with collimator setting of 15° (V2_C15_) was superior to double arc with collimator 60° (V2_C60_) in 6 out of 20 (30%) cases. Similar plan quality was achieved in 11 cases. The Wilcoxon test showed that plan quality differed significantly (*p* = 0,0008 and *p* = 0,008) for both evaluation methods (scoring and COV based) between V2_C15_ and V2_C60_. No significant difference was found comparing V2_C15_ with V2_C40_. Plan quality of V2_C15_ was superior in 7 out of 20 (35%) cases while V2_C40_ was better in 6 out of 20 (30%) cases. As shown in Table 6, larger deviations in delivery time and conformity index were observed for comparison 5 (V2_C15_ vs. V2_C60_). The decrease of conformity was 0.5% on average in comparison 4 (V2_C15_ vs. V2_C40_).

In comparison 6 and 7, the two collimator settings (2V1_C15_60_ and 2V1_C15_345_) were compared to the reference plan (V2_C15_). Plan quality of V2_C15_ differed significantly from 2V1_C15_345_. V2_C15_ was superior in 14 of 20 cases. Plan metrics differed by − 1,5, 29% and − 1,5% for monitor units, delivery time and conformity index (Table 6). The Wilcoxon test showed no significant difference between 2V1_C15_60_ and V2_C15_. The change of monitor units was − 1,5% on average. The conformity index deviated by − 0,4%. Similar plan quality could be achieved for 2V1_C15_60_ and V2_C15_.

Additionally, the two single arcs were compared with each other in comparison 8 (Table 5). 2V1_C15_60_ was superior against 2V1_C15_345_ in 14 of 20 cases.

**Fig. 3:**
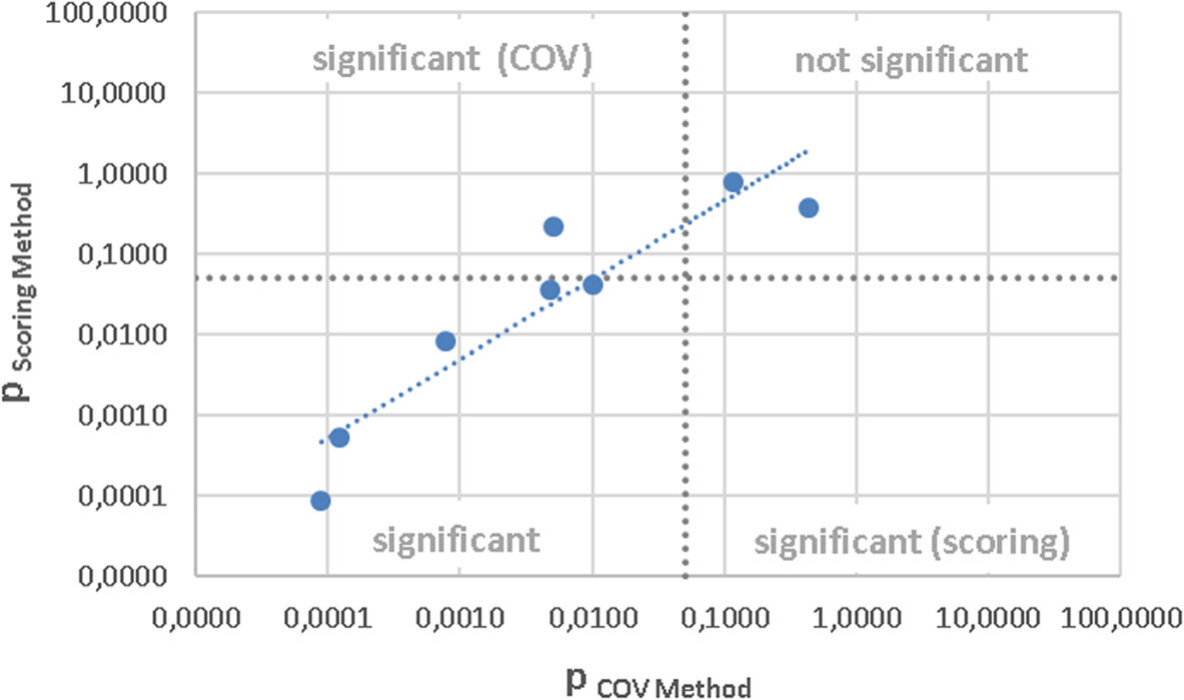
Comparison of the scoring method and COV method. The correlation of *p*-values derived from Wilcoxon test is plotted in logarithmic scaling. Significance level (dashed line) and corresponding quadrants are shown

The correlation of scoring and COV method is shown in Fig. 3. The *p*-values from the Wilcoxon test are plotted in logarithmic scaling. A strong correlation between the two evaluation methods was found with a correlation coefficient of 0,91 and a higher sensitivity of the COV method.

## Discussion

In this work, it was investigated which VMAT beam configuration shows superior plan quality in head and neck cancer patients. The double arc technique resulted in an improved plan quality compared to one single arc or two single arcs. This superiority of double arc VMAT plans compared with single arc in terms of PTV coverage and OAR sparing was also confirmed in other planning studies for head and neck cases [14–17]. Guckenberger et al. showed that multiple arc VMAT improved the plan quality compared to single arc VMAT at cost of increased delivery times, increased monitor units and increased spread of low doses [14]. Tol et al. have quantitatively demonstrated increased plan quality when more than two arcs are used. They concluded that the four arc plans seemed to provide a good trade-off between increased delivery time and improved plan quality [15].

We investigated if there is a difference in plan quality using double arc or two single arcs. Plan quality was superior for the double arc technique for the same collimator setting. This may be due to how the two arcs are handled during optimization and sequencing. As already suggested by Yang et al., the definition of two single arcs before optimization allows the user to configure the collimator position of the second arc different from the first arc [18]. The collimator position is an additional setup parameter which can further improve plan quality. For the current investigation, it was found that a collimator setting of 15° and 60° should be preferred to a collimator setting of 15° and 345° when two single arcs are used.

Similar plan quality and smallest deviation of conformity was observed for a double arc technique with collimator setting 40° (V2_C40_) and for a two single arc technique with collimator setting of 15° and 60° (2V1_C15_60_). The superior collimator position potentially depends on the individual target volume shape and complexity. If the relationship between target volume complexity and corresponding superior beam arrangement would be known, the superior beam arrangement could be chosen based on the complexity index of the target volume. This can potentially save time during the treatment planning process.

The current investigation of the arc length showed that full arcs were superior to partial arcs. Contrary to expectation for using partial arcs, the irradiation across the ipsilateral side and sparing of the healthy side would provide benefits; the full rotation is the superior technique. Perhaps, this is due to the additional degree of freedom. In previous studies different arrangements of full and partial arcs were compared for the treatment of head and neck cases [18, 19]. Miura et al. found that partial arcs were comparable with the full-arc plans regarding dose homogeneity and conformity in maxillary cancer and provided a statistical decrease in mean dose to OAR, total MU, delivery time and gantry angle error [19]. Yang et al. stated that it will be challenging to generate partial arc plans for complicated cases [18].

Furthermore, a new cross comparison method was introduced in this study to allow a simple comparison for plans that use different sets of assessment scales, derived during the optimization process of each plan. Its results were similar to that of a classical method of scoring of an evaluation parameter set; cross comparison seems to be applicable especially for Auto-Planning settings. The COV method is more sensitive than the scoring method.

### Limitations

The current work was limited to an evaluation of single and double arc techniques based on patient cases for head and neck cancer. Further improvement of plan quality would be expected for using a larger number of arcs or non coplanar beam arrangements. Multiple arc VMAT showed improved plan quality compared to single arcs at cost of increased delivery times and monitor units [14, 15].

As Teoh et al. already mentioned, direct comparisons between different studies are difficult because of significant differences in target volume definitions, target complexity, dose prescription and treatment schedules [20]. An additionally impact will have the treatment planning system and the implemented algorithms for VMAT optimization and segmentation.

## Conclusion

The impact of different beam configurations on plan quality using Pinnacle^3^ Auto-Planning module was investigated in this study. Quantitative evaluation showed that double arc and full rotation are superior to single arc and partial rotation techniques in terms of organs at risk sparing even for unilateral target volumes. The collimator position was found as an additional setup parameter which can further improve the target coverage and sparing of organs at risk.

## Abbreviations

2V1_CA_CB_: Two single arcs with different collimator positions (A and B)
CI: Conformity Index
COV: Composite objective value
DVH: Dose volume histogram
OAR: Organ at risk
PTV: Planning target volume
S: Set of objectives
SIB: Simultaneous integrated boost
T: Delivery Time
V1_C15_: Single arc with collimator 15°
V2_C15_: Full double arc with collimator 15°
V2_C15_Part_: Partial arc with collimator 15°
VMAT: Volumetric modulated arc therapy
